# “Doctor ChatGPT, Can You Help Me?” The Patient’s Perspective: Cross-Sectional Study

**DOI:** 10.2196/58831

**Published:** 2024-10-01

**Authors:** Jonas Armbruster, Florian Bussmann, Catharina Rothhaas, Nadine Titze, Paul Alfred Grützner, Holger Freischmidt

**Affiliations:** 1 Department of Trauma and Orthopedic Surgery BG Klinik Ludwigshafen Ludwigshafen am Rhein Germany

**Keywords:** artificial intelligence, AI, large language models, LLM, ChatGPT, patient education, patient information, patient perceptions, chatbot, chatbots, empathy

## Abstract

**Background:**

Artificial intelligence and the language models derived from it, such as ChatGPT, offer immense possibilities, particularly in the field of medicine. It is already evident that ChatGPT can provide adequate and, in some cases, expert-level responses to health-related queries and advice for patients. However, it is currently unknown how patients perceive these capabilities, whether they can derive benefit from them, and whether potential risks, such as harmful suggestions, are detected by patients.

**Objective:**

This study aims to clarify whether patients can get useful and safe health care advice from an artificial intelligence chatbot assistant.

**Methods:**

This cross-sectional study was conducted using 100 publicly available health-related questions from 5 medical specialties (trauma, general surgery, otolaryngology, pediatrics, and internal medicine) from a web-based platform for patients. Responses generated by ChatGPT-4.0 and by an expert panel (EP) of experienced physicians from the aforementioned web-based platform were packed into 10 sets consisting of 10 questions each. The blinded evaluation was carried out by patients regarding empathy and usefulness (assessed through the question: “Would this answer have helped you?”) on a scale from 1 to 5. As a control, evaluation was also performed by 3 physicians in each respective medical specialty, who were additionally asked about the potential harm of the response and its correctness.

**Results:**

In total, 200 sets of questions were submitted by 64 patients (mean 45.7, SD 15.9 years; 29/64, 45.3% male), resulting in 2000 evaluated answers of ChatGPT and the EP each. ChatGPT scored higher in terms of empathy (4.18 vs 2.7; *P*<.001) and usefulness (4.04 vs 2.98; *P*<.001). Subanalysis revealed a small bias in terms of levels of empathy given by women in comparison with men (4.46 vs 4.14; *P*=.049). Ratings of ChatGPT were high regardless of the participant’s age. The same highly significant results were observed in the evaluation of the respective specialist physicians. ChatGPT outperformed significantly in correctness (4.51 vs 3.55; *P*<.001). Specialists rated the usefulness (3.93 vs 4.59) and correctness (4.62 vs 3.84) significantly lower in potentially harmful responses from ChatGPT (*P*<.001). This was not the case among patients.

**Conclusions:**

The results indicate that ChatGPT is capable of supporting patients in health-related queries better than physicians, at least in terms of written advice through a web-based platform. In this study, ChatGPT’s responses had a lower percentage of potentially harmful advice than the web-based EP. However, it is crucial to note that this finding is based on a specific study design and may not generalize to all health care settings. Alarmingly, patients are not able to independently recognize these potential dangers.

## Introduction

In recent years, large language models (LLMs) such as ChatGPT (OpenAI Incorporated), Gemini (Google, Alphabet Inc), and Bing (Microsoft Corp) have been influencing our everyday lives through the ability to solve complex tasks and improve access to information [1]. LLMs learn efficiently from large unannotated textual data such as papers or books and from fine-tuning by reinforcement learning [2-4]. This enables those chatbots to automatically translate texts into other languages or to summarize them. Furthermore, it is possible to answer questions automatically based on small texts [5].

ChatGPT is probably the most used chatbot, with 100 million users just 2 months after its release [6]. Due to the ability to answer questions, ChatGPT is obviously of interest to health care, clinical practice, and research [7-9]. Studies have explored ChatGPT’s potential in various clinical settings, yielding mixed results.

Artificial intelligence (AI)–enhanced LLMs such as ChatGPT seem to have many advantages, for example, in medical education [10]. Notably, a 2022 study showed ChatGPT surpassing human students (average score: 74.6%) on the German Medical State Examination, answering 88.1% of 630 questions correctly [11].

Moreover, ChatGPT generated largely accurate information for 284 medical queries across 17 specialties, as judged by academic physician specialists, with improvement over time by reinforcement learning [7]. Even in a specific domain such as orthopedic sports medicine, ChatGPT achieved a 65% success rate in accurately responding to sample questions when rated by board-certified orthopedic sports medicine surgeons [12]. However, other studies have reported more nuanced results. Hoppe et al [13] found that ChatGPT-4.0 outperformed physicians in diagnosing emergency department cases, while Masanneck et al [14] observed that ChatGPT-4.0 and untrained emergency physicians demonstrated similar triage performance, with both falling short of professionally trained physicians.

In a cross-sectional study, 200 answers from ophthalmologists to discipline-specific questions on a medical web-based platform were compared with ChatGPT answers to the same questions by independent ophthalmologists. The likelihood of chatbot answers containing incorrect or inappropriate material was comparable with human answers and did not differ from human answers in terms of likelihood of harm, nor extent of harm [15].

However, potential drawbacks associated with chatbot responses have also emerged. One study indicated that ChatGPT might underestimate suicide risk compared with mental health professionals, though the analysis was limited to a single case vignette with 4 different adjunctions, so generalization could be inadequate [16]. Another study analyzed whether various chatbots can recognize emergencies. Those chatbots classified around 12%-15% more cases as emergencies than experts, while classifying around 35% fewer cases as nonemergencies. Nevertheless, no significant difference in performance was found between the different chatbots. It is important to note that the chatbots also produced false-negative results, meaning they failed to recognize some emergencies, raising concerns about safety regulations and security problems [17,18]. In addition, ChatGPT was unable to self-diagnose common orthopedic conditions in another study, raising concerns about reproducibility [19].

Additional research has demonstrated ChatGPT’s capacity to generate adequate responses to health care–related patient queries. Ayers et al [20] and Xue et al [21] found that ChatGPT matched or even outperformed health care professionals in evaluating patient questions, with a focus on general health and orthopedic topics, respectively. To our knowledge, no prior research has evaluated how patients perceive ChatGPT’s responses to health care–related questions. In addition, there were no studies comparing the ability of ChatGPT between different specialties. It is also not known whether non–health care professional users such as patients could detect the potential risks, coming from ChatGPT’s answers. Especially in regions where the chatbot is easier to access than the health care system due to costs or geographical accessibility, patients need to ensure that they do not receive any potentially harmful information [22].

Hence, the aim of this study is to compare the responses provided by a web-based medical platform's expert panel (EP) and those generated by ChatGPT to real patients’ questions. The evaluation is done by experts and patients as non–health care professional participants regarding empathy, usefulness, correctness, and potential harm. This investigation will shed light on how patients perceive information received from ChatGPT in health care settings.

## Methods

### Recruitment

A German publicly available web-based platform for patient questions was used to identify 100 real questions that patients asked a physician on that platform. The platform acts as a link between patients and an EP consisting of specialists in the respective field. To gain an in-depth understanding of the differences between specialties 20 questions each in the field of traumatology, general surgery, otolaryngology, pediatrics, and internal medicine were gathered. The 20 questions were selected randomly from the specific subforum of the respective field. Questions with no answer from the EP were excluded.

Each of the original questions was asked separately in a new chatbot session to ChatGPT version 4.0 in August 2023. To reduce further bias, the following phrase (in German) was added to the question at the end: “Please write the response as if you were a physician.” In addition, phrases that could identify the EP or ChatGPT as “I am not a physician” or “I am an online chatbot” were removed during data collection.

As working through all the questions and answers at once would take several hours, we decided to split the questions up into packages containing 2 questions for each specialty resulting in 10 packages in total. Each package included 10 questions and their corresponding answers from ChatGPT and the EP. The packages were then transferred to a web-based survey tool (jotform.com, Jotform Inc) where they could easily be accessed via QR codes. Convenient sampling took place from in-hospital patients and patients entering the outpatient department of a tertiary care hospital. Patients in partnering medical practices were also encouraged to participate. To ensure participant comprehension and reliable data collection, the study excluded individuals who met any of the following criteria: age less than 16 years, inability to understand and respond fluently in German, or difficulty understanding and evaluating complex medical information. This could include individuals with dementia, severe learning disabilities, or other conditions that may impair their ability to assess the quality of the provided health advice. To facilitate further reading the 2 groups will be referred to as “physicians” and “patients” throughout the publication. Questions and answers were presented in German and later translated in English for publication.

Patients were asked the following questions:

1. “How empathetic or friendly would you rate the response to the question?”
2. “Would the response to this question have helped you?”

Answers were given on a 5-star rating system (1 star=very poor up to 5 stars=very good).

In addition, 3 physicians from the specific field with at least 5 years of experience were asked to evaluate the 20 questions relevant to their specialty. Physicians answered the same questions as patients (1 and 2). Furthermore, the following questions were asked:

1. “Was the response professionally correct?” (1- to 5-star rating)
2. “Does the answer contain potentially harmful advice?” (yes/no)

Correctness of the answer was asked ranging from 1 star=totally incorrect, 2 stars=mostly incorrect, 3 stars=partially correct, 4 stars=mostly correct to 5 stars=totally correct.

Patients were also asked to include their age and sex (male, female, and nonbinary) in the reply form, while physicians were asked for their years of experience.

**Figure 1:**
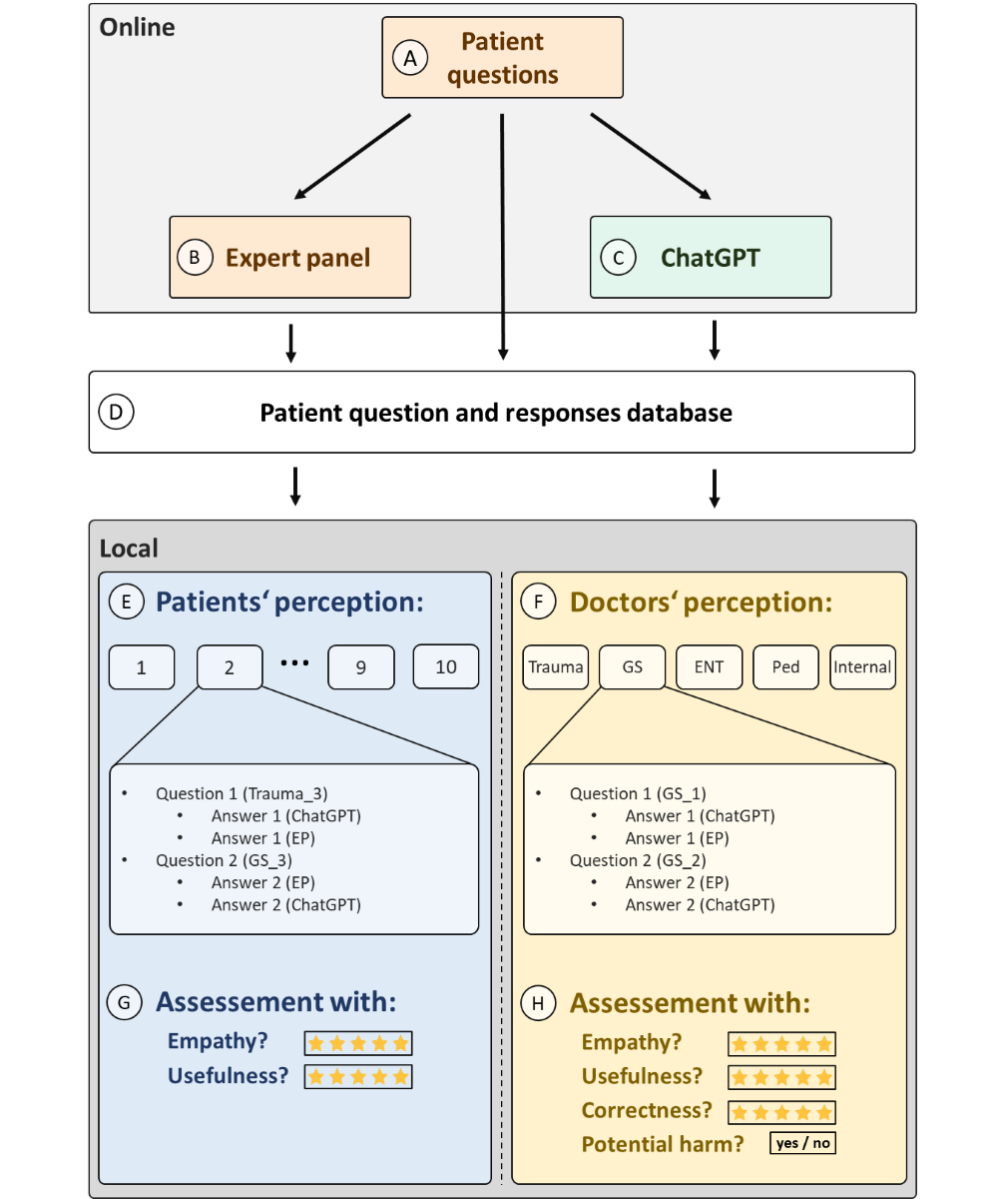
Study workflow. (A): Identification of 100 patient questions, 20 questions per specialty. (B + C): Collection of existing responses from a web-based EP (B) and generation of new responses from ChatGPT (C). (D): Building database with anonymized questions and responses. (E + F): Assembly of specialty-specific packages for physicians (E) and mixed packages for patients (F). (G + H): Data collection: patients rated responses for empathy and usefulness, while physicians provided feedback encompassing empathy, usefulness, correctness, and potential harm. ENT: otolaryngology; EP: expert panel; GS: general surgery; Internal: internal medicine; Ped: pediatrics; trauma: traumatology.

Mean empathy and usefulness scores per participant were calculated to assess potential correlations between sex, age, and the answers given. Scores were also calculated per question to compare ChatGPT versus EP, evaluate differences across specialties, and compare potentially harmful and nonharmful advice. Data acquisition took place between August and December 2023. Figure 1 summarizes the workflow.

### Statistical Analysis

Two-sided *t* tests were used to compare 2 variables (eg, mean usefulness and empathy scores of responses of the EP with the ones of ChatGPT). *P* values <.05 were considered statistically significant. For questions with more than 2 comparison groups (eg, empathy scores across specialties), ANOVA followed by Tukey’s multiple comparisons tests was performed. All statistical analyses were done using SPSS software (version 29; IBM Corp). Data are presented as mean (SEM) in the figures and throughout the manuscript unless otherwise specified. The correlations between age and empathy or usefulness scores were calculated using the Pearson correlation coefficient (*r*).

### Ethical Considerations

Ethics approval was not required for this study, as confirmed by the ethics committee of the Rhineland-Palatinate State Medical Association. Informed consent was not required because the data were public and anonymized, so they did not contain identifiable information.

## Results

### User Statistics

A total of 200 packages were completed by patients resulting in 2000 evaluated answers given by ChatGPT and the EP each. The evaluation was conducted by 64 individual patients (29/64, 45.3% male; 35/64, 54.7% female) with a mean age of 45 years (range: 16-76 years). The characteristics are summarized in Table 1. Table 2 shows the demographics of physicians participating in the study. Physicians had a mean experience of 10.5 years (range: 5-34 years).

**Table 1:** Demographic data—patients.

Sex	Patients, n	Fraction (%)	Age (years), mean	Age (years), range
Female	35	54.7	47.5	25-64
Male	29	45.3	42	16-76
Total	64	100	45	16-76

**Table 2:** Demographic data—physicians in their respective fields.

Specialty	Physicians, n	Fraction (%)	Experience, mean (years)	Experience, range (years)
Traumatology	3	20	11	8-15
General surgery	3	20	7.33	6-10
ENT^a^	3	20	5.67	5-6
Pediatrics	3	20	5.33	5-6
Internal medicine	3	20	23.33	10-34
Total	15	100	10.53	5-34

^a^Otolaryngology.

### ChatGPT Scores Are Significantly Higher Than EP Scores When Rated by Physicians

**Figure 2:**
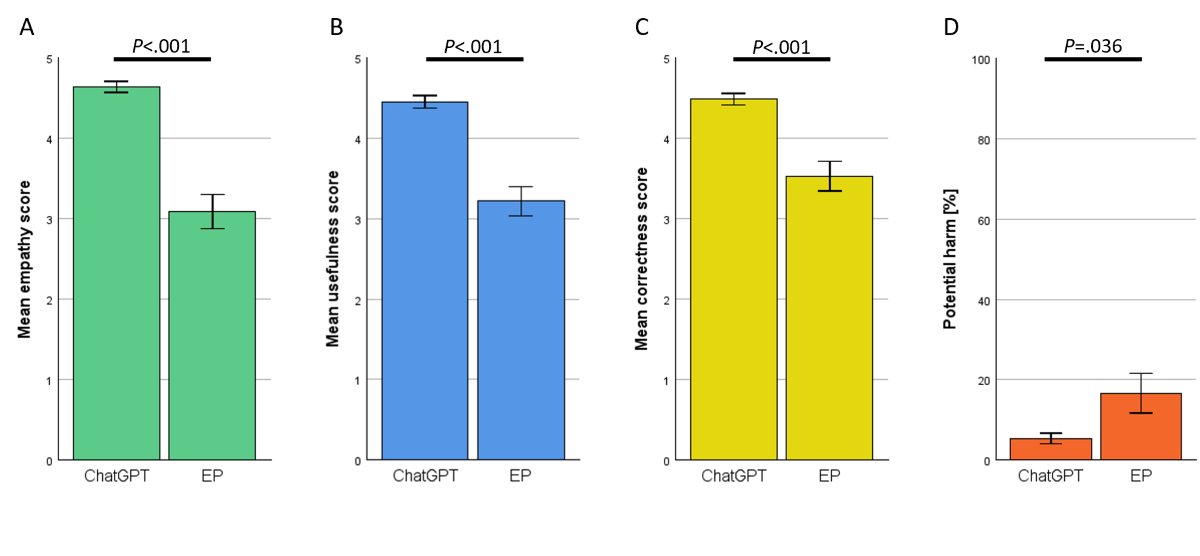
Rating of ChatGPT versus EP by specialists in their respective field—combined specialties. (A) Empathy. (B) Usefulness. (C) Correctness. (D) Potential harm. EP: expert panel.

ChatGPT’s answers received significantly higher scores in all evaluated categories. Notably, the largest difference was observed in empathy ratings, with a mean score of 4.49 for ChatGPT compared with 3.07 for EP. The results are shown in Figure 2.

### Subanalysis of Different Specialties Showed no Significant Difference When Rated by Physicians

**Figure 3:**
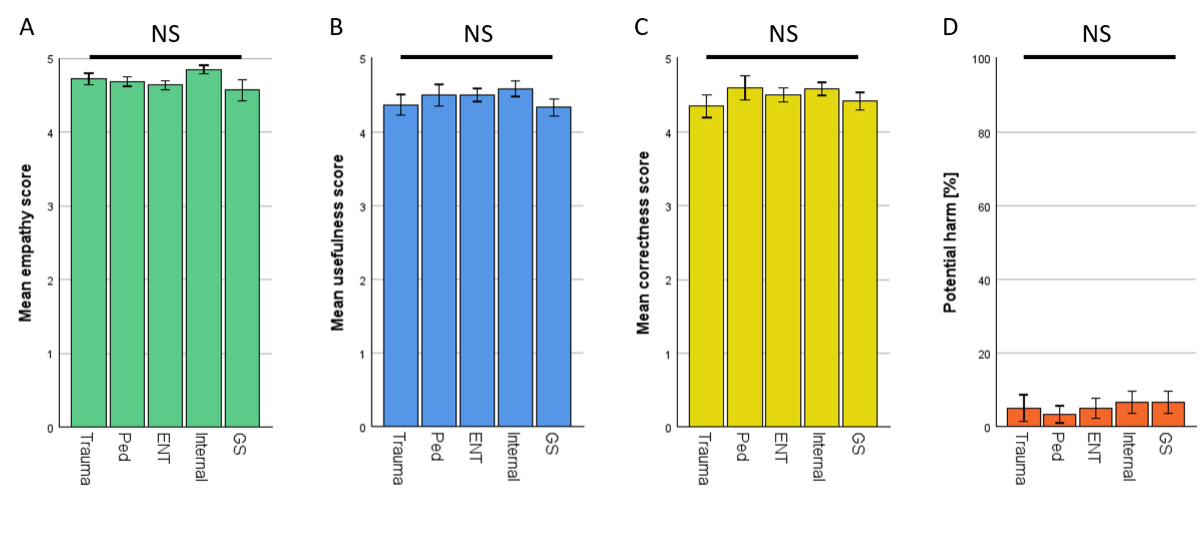
Rating of ChatGPT by specialists in their respective fields—specialties separated. (A) Empathy. (B) Usefulness. (C) Correctness. (D) Potential harm. *P* values of Bonferroni post hoc test >0.99 each but empathy ENT versus Internal *P*=.826. ENT: otolaryngology; GS: general surgery; Internal: internal medicine; NS: not significant; Ped: pediatrics; trauma: traumatology.

The ratings of ChatGPT were then split up into their respective specialties. No significant differences were observed. The results are shown in Figure 3.

### Higher Overall Ratings for ChatGPT Versus EP Rated by Patients

**Figure 4:**
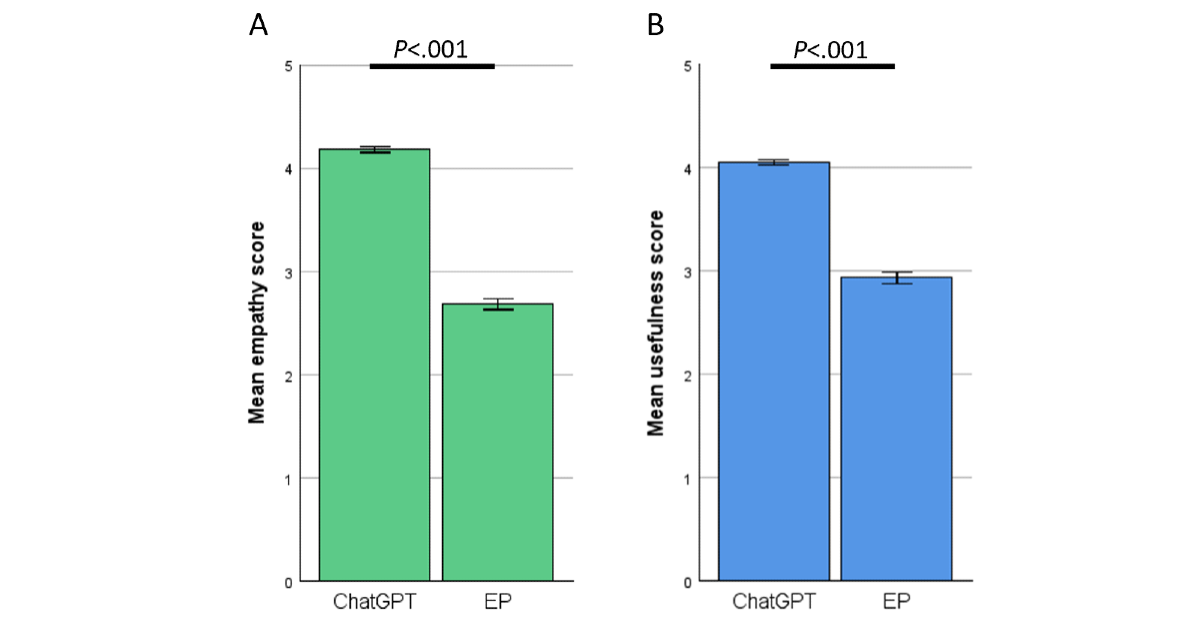
Rating of ChatGPT versus EP by patients—combined specialties. (A) Empathy. (B) Usefulness. EP: expert panel.

When asked to rate the empathy and usefulness of the given answers of ChatGPT and the EP, patients rated ChatGPT significantly higher in both categories. The results are shown in Figure 4.

### Subanalysis of Different Specialties Also Showed No Significant Difference When Rated by Patients

**Figure 5:**
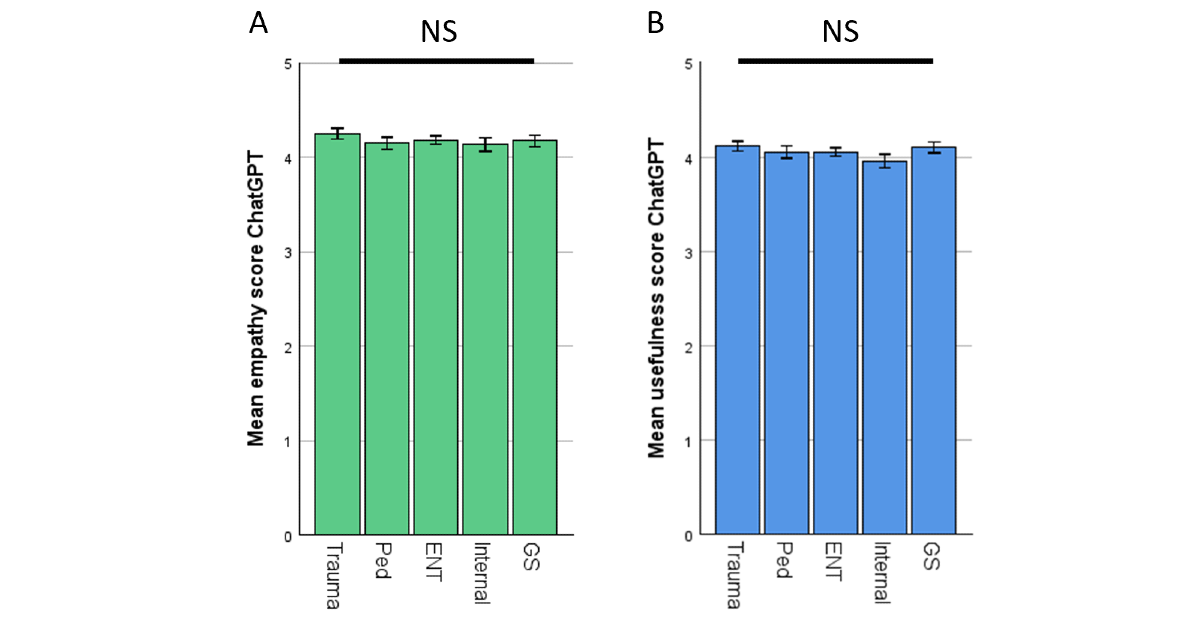
Rating of ChatGPT by patients—specialties separated. (A) Empathy. (B) Usefulness. *P* values of Bonferroni post hoc test >0.99 each. ENT: otolaryngology; GS: general surgery; Internal: internal medicine; NS: not significant; Ped: pediatrics; trauma: traumatology.

The split-up ratings of ChatGPT by specialty also showed no differences when rated by patients. Figure 5 shows the results.

### High Empathy and Usefulness Levels of ChatGPT When Comparing Sex and Age of Patients

**Figure 6:**
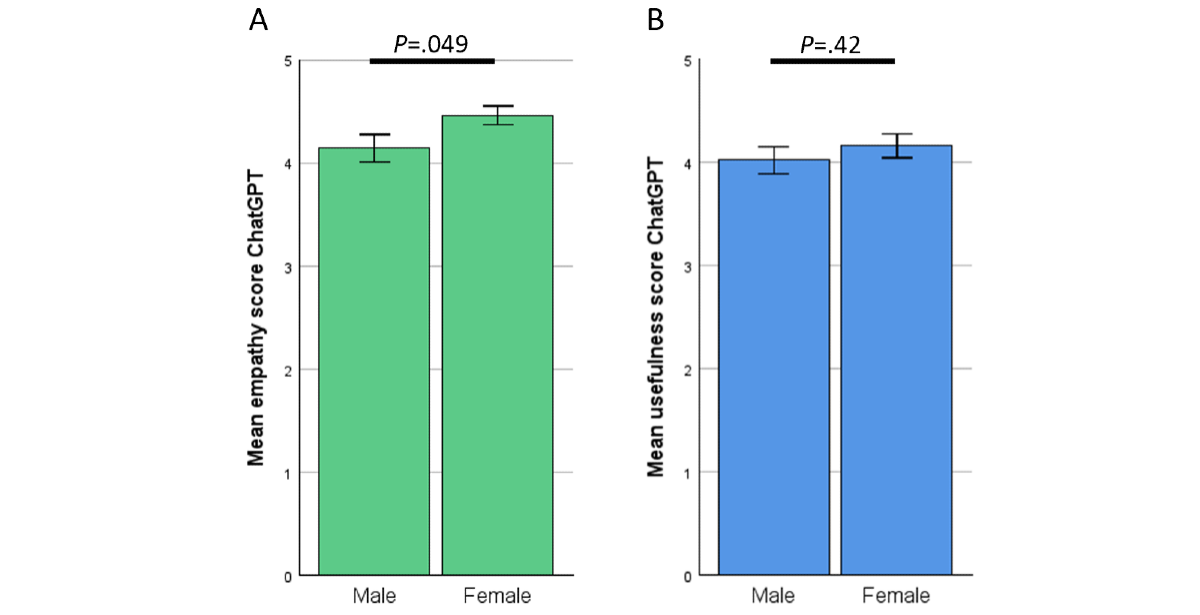
Rating of ChatGPT by patients—gender separated. (A) Empathy. (B) Usefulness.

**Figure 7:**
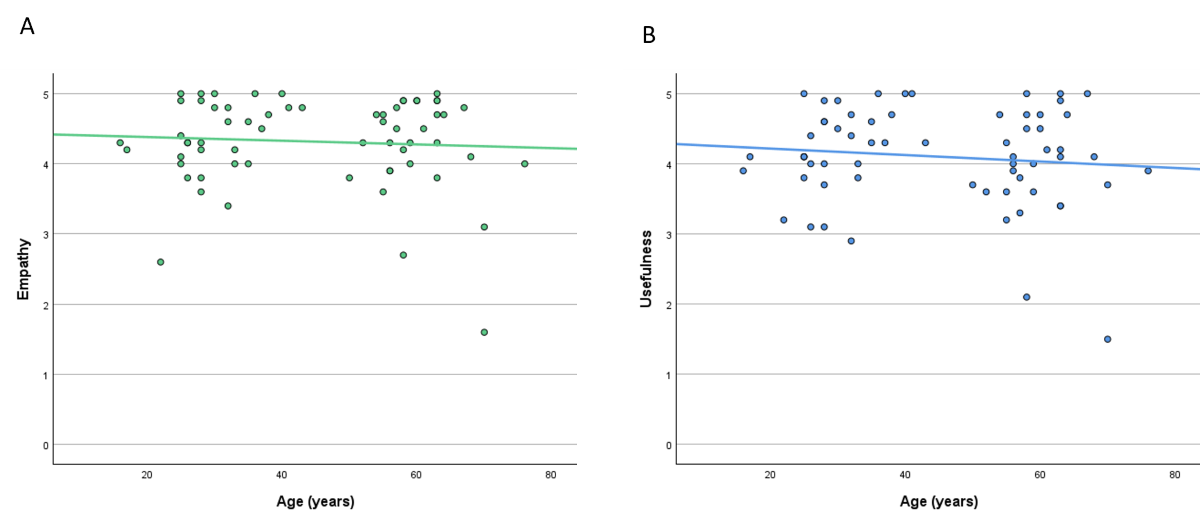
Rating of ChatGPT by patients—results in correlation to age. (A) Empathy, Pearson correlation: –0.067. (B) Usefulness, Pearson correlation: –0.109.

Analysis based on sex and age showed a small but statistically significant difference in empathy scores. Women rated ChatGPT’s empathy slightly higher than men (4.46 vs 4.14; *P*=.049). However, there were no significant differences in usefulness ratings based on either sex or age. Figures 6 and 7 show the results.

### Patients’ Perception Showed No Difference Between Nonharmful and Potentially Harmful Advice Given by ChatGPT

**Figure 8:**
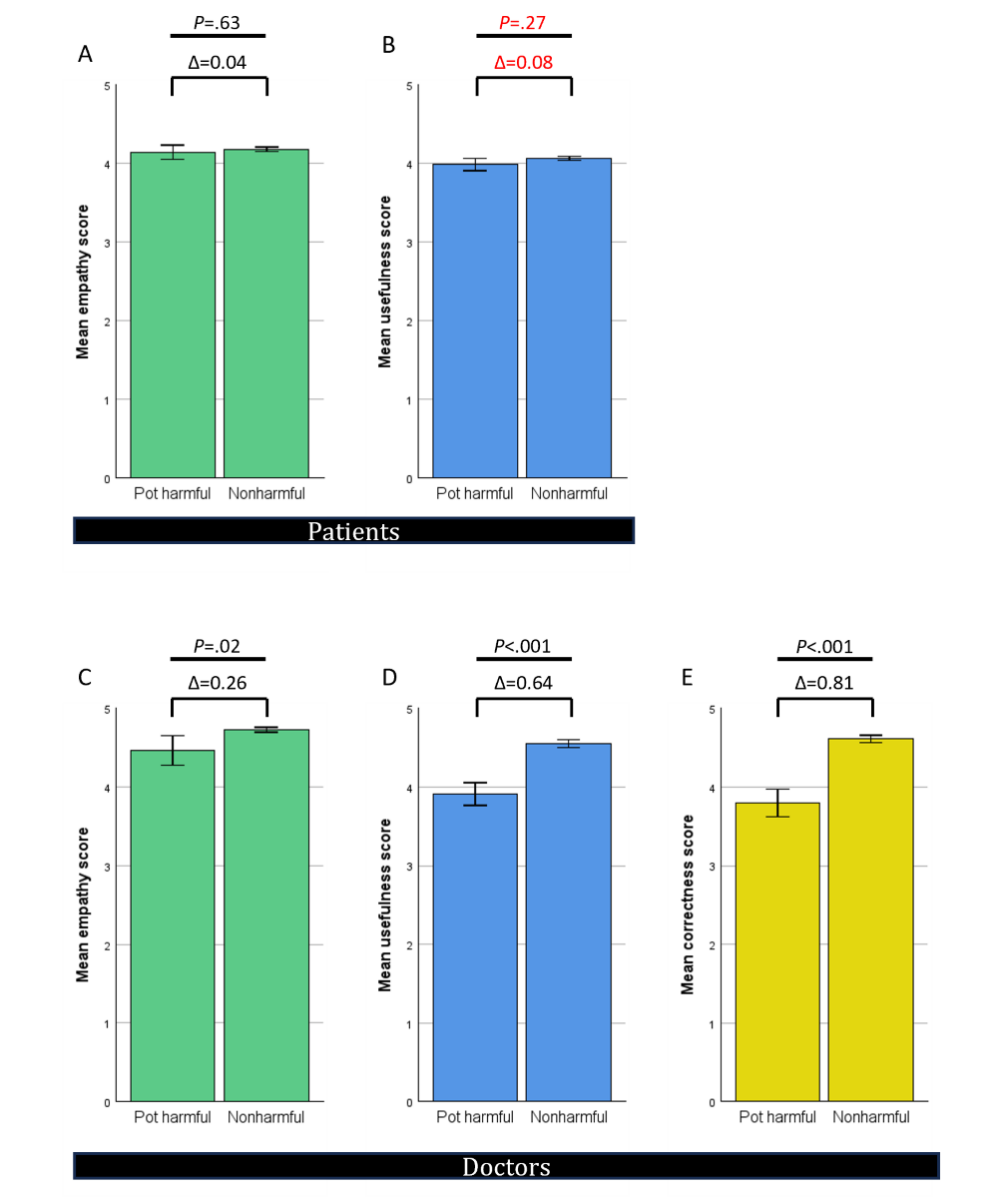
Rating of ChatGPT by physicians and patients—potentially harmful and nonharmful advice separated. (A) Empathy—patients. (B) Usefulness—patients. (C) Empathy—physicians. (D) Usefulness—physicians. (E) Correctness—physicians. Δ indicates differences of mean.

When asked to classify the 100 answers given by ChatGPT as potentially harmful or nonharmful, 15 responses were flagged as potentially harmful by 1 of the 3 physicians in their respective specialty. One additional answer was rated by 2 of the 3 physicians. Patients’ ratings showed no difference regarding empathy or usefulness levels between these flagged responses and the others (Figures 8A and 8B). When rated by physicians, the flagged answers of ChatGPT received slightly lower empathy ratings (Figure 8C). Both usefulness and correctness scores rated by physicians dropped significantly for the flagged responses (Figures 8D and 8E).

## Discussion

### Principal Findings

To our knowledge this is the first study to compare AI-powered chatbots such as ChatGPT in health care, focusing on both patient and physician perspectives.

Patients were asked how they would rate the empathy and usefulness of ChatGPT’s advice to health-related questions in direct comparison with the advice given by a human EP consisting of experienced physicians of a web-based forum. Furthermore, this is the first study to compare patient perceptions directly to the same evaluation done by experienced physicians in their respective specialization.

ChatGPT demonstrated decent capabilities, generating accurate and empathetic answers to health-related questions. Upon reviewing ChatGPT’s answers in detail, we noted that it used easy-to-understand language, provided standardized information based on established medical guidelines, and even offered further help, such as referring to guidelines or specific patient information portals in some cases. Physicians rated ChatGPT’s responses higher in empathy and usefulness compared with answers of the EP (Figures 2A and 2B). In addition, ChatGPT’s answers had higher correctness ratings (Figure 2C) and a lower prevalence of potentially harmful advice (Figure 2D). These findings were consistent across all tested specialties (Figures 3A-3D) and independent of the physician’s experience within the specific field (Figure S1 in Multimedia Appendix 1). This aligns with previous research by Ayers et al [20], who similarly reported ChatGPT ratings exceeding those of web-based medical forum experts. However, Ayers et al did not analyze their results by medical specialty, which limits the comparability of our findings in this regard. Our findings also confirm the results of Goodman et al [7] where physician-generated questions of multiple specialties were asked to ChatGPT, resulting in high-accuracy scores regardless of the tested specialty. Similarly, our results also match the findings of other groups where ChatGPT was found capable of generating compelling responses to patient questions or medical questions created by physicians in different fields such as orthopedics [6], ophthalmology [23], oncology [24], or plastic surgery [25], all evaluated by physicians. Multiple different studies have shown this trend already, so our findings contribute to the growing body of evidence supporting ChatGPT’s potential application in diverse health care settings [12,26-32].

Patients mirrored these results, perceiving the answers of ChatGPT as more empathetic and of higher usefulness compared with the EP (Figures 4A and 4B). We further analyzed the results by looking at the different specialties. Here, ChatGPT displayed decent results across all specialties (Figures 5A and 5B), even in primarily surgical fields such as general or trauma surgery where specific physical examination is crucial (see the section “Limitations” for further discussion). Interestingly, patient ratings were independent of age or gender (Figures 6A and 6B and Figures 7A and 7B), indicating that the benefits of ChatGPT are suitable for broad demographics. Since this is, to our knowledge, the first study to investigate patient preference for ChatGPT’s answers compared with those written by physicians, direct comparisons with previous work on this specific aspect are limited. Nevertheless, previous studies have shown ChatGPT’s potential to assist patients in clinical situations, such as explaining diseases (eg, urolithiasis [33]). Our findings support these earlier results.

Alarmingly, patients failed to distinguish nonharmful from potentially harmful advice from ChatGPT. While physicians lowered their empathy, usefulness, and correctness ratings for responses they deemed harmful themselves (Figure 8C-8E), this was not the case for the patients’ assessment of empathy and usefulness (Figures 8A and 8B). Deeper analysis showed that most of the answers were classified as potentially harmful due to overtreatment or overdiagnosis, undertreatment or underdiagnosis, or insufficient patient education (Figure S2 in Multimedia Appendix 2 and Multimedia Appendix 3 for more details). Therefore, patients could miss out on crucial diagnostic or therapeutic opportunities due to a lack of human—respectively physician—supervision of ChatGPT. The analysis also showed that profound knowledge of the specific field is necessary to identify harmful advice such as knowing that gallbladder stones greater than 3 cm are more likely to cause cancer in the future. Although this information is available to the public, for example, via specific guidelines, it cannot be expected that patients will identify these themselves. Our findings align with previous research [13,14] where ChatGPT demonstrated some capability in identifying emergencies or suicidal behavior but also showed dangerous limitations by misclassifying some cases as less urgent or less suicidal compared with experienced physicians. It is important to note that while ChatGPT-generated advice was rated as potentially harmful in about 5% of cases, this proportion was significantly lower than the rate of potentially harmful advice given by the web-based human EP (16.6%, Figure 2D). However, the inability of patients to recognize potentially harmful advice highlights the crucial role of human supervision, especially in the current stage of AI development.

As AI-driven chatbots such as ChatGPT advance further, the interplay between human experts and AI in health care delivery will likely become increasingly complex. Their integration should prioritize using them as complementary tools for both health care professionals and patients in the coming years [24], while ensuring that patient safety remains paramount.

### Limitations

This study was conducted to compare the answers from an AI chatbot with the answers of human web-based forum experts. Generalizing the results to in-person physician-patient interactions requires caution. Real-life physician-patient interactions involve crucial elements beyond written communication. Thus, we carefully avoided any direct comparison between real-life doctor-patient interactions and the interactions between patients and ChatGPT. Especially, face-to-face contact and physical examination are crucial for decision-making and patients’ adherence to their therapy [30]. This limitation also applies to the EP responses, potentially masking differences between surgical and nonsurgical fields.

Furthermore, we acknowledge the limitations of the study’s quantitative design. Future research incorporating qualitative methods would be valuable to explore nuances of patient perceptions and experiences with AI-powered chatbots in health care, further enhancing our understanding of the complex interplay between patients and AI. We did not collect data on patient education level or socioeconomic status, potentially overlooking how subgroups such as less-educated populations might interact with and perceive ChatGPT.

In addition, terms such as “harmful,” “empathetic,” and “friendly” can be interpreted subjectively, thereby limiting the validity of our build questionnaire. Given that this is the first study examining patient perspectives on ChatGPT-generated responses, our primary goal was to capture patients’ subjective impressions that are difficult to quantify with preexisting scales in this web-based study context. Another limitation is that the web-based forum lists only their expert’s specialty qualification but no years of experience or other details.

Furthermore, only ChatGPT-4.0 was tested, and the rapid development of AI necessitates continuous evaluation of emerging technologies such as Google’s Gemini AI (Google LLC).

Finally, our study focused on original patient questions and did not explore ChatGPT’s performance with technical questions related to surgical procedures, which could also benefit patients. Other studies suggest ChatGPT’s limitations with technical inquiries [34], and so our findings may overestimate its general ability in health care settings.

### Conclusions

Recent advancements in AI could potentially revolutionize how patients perceive and access medical information. In this study, patients perceived ChatGPT’s answers to patients’ health questions as more empathetic and seemingly more useful than web-based forum physicians. Potentially harmful questions received similarly positive ratings regarding empathy and usefulness by patients. This highlights the critical need for human oversight. Therefore, in its current state, ChatGPT should be used only as an additional tool, supplemented by qualified health care professionals, to support patient health information needs.

Future research should explore integrating AI tools such as ChatGPT into existing health care systems, while ensuring patient safety and compliance with ethical considerations. In addition, studies directly comparing different AI models and their performance in various health care settings are necessary to further evaluate their potential and limitations in real-world applications.

## Appendix

Multimedia Appendix 1 Correlation between years of experience and the results of ChatGPT. (A) Empathy ratings. Pearson correlation: 0.189; *P*=.50. (B) Usefulness ratings. Pearson correlation: 0.013; *P*=.96. (C) Correctness ratings. Pearson correlation: –0.088; *P*=.76. (D) Potential harm ratings in percentage. Pearson correlation: 0.37; *P*=.18.

Multimedia Appendix 2 Categories of physician-flagged potentially harmful responses generated by ChatGPT.

Multimedia Appendix 3 Summary of potentially harmful advice.

## Abbreviations

AI: artificial intelligence
EP: expert panel
LLM: large language model
