# Supplementary figures and images for: “Doctor ChatGPT, Can You Help Me?” The Patient’s Perspective: Cross-Sectional Study
Source: J Med Internet Res. 2024 Oct 1;26:e58831. doi: 10.2196/58831 (PMC11480680; doi:10.2196/58831)

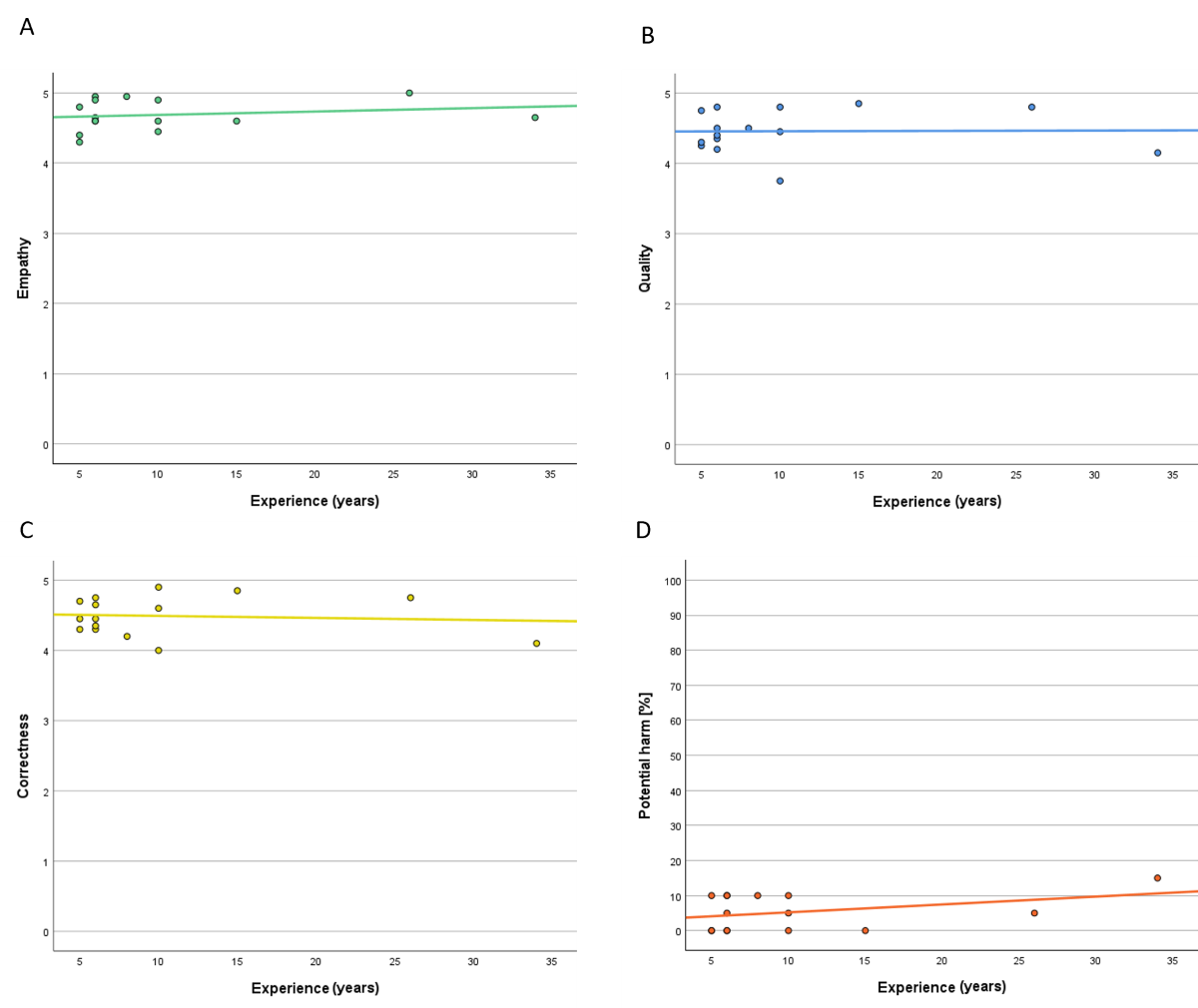

Supplement: Multimedia Appendix 1 [file jmir_v26i1e58831_app1.png]

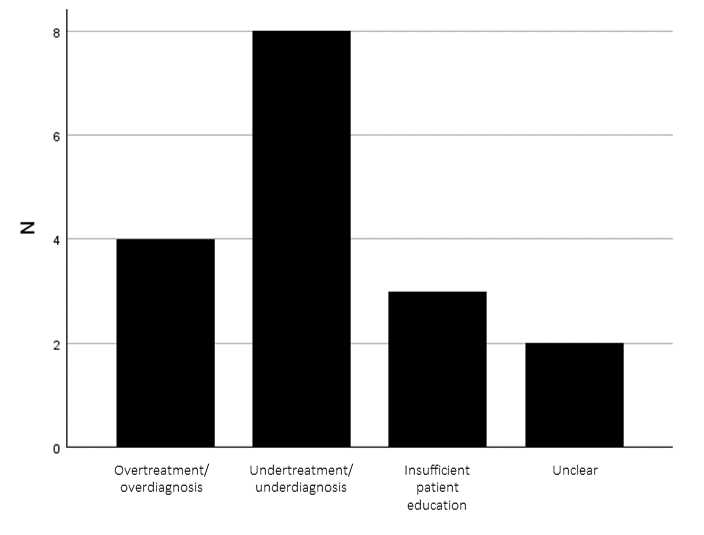

Supplement: Multimedia Appendix 2 [file jmir_v26i1e58831_app2.png]
