# Supplementary material for: “Doctor ChatGPT, Can You Help Me?” The Patient’s Perspective: Cross-Sectional Study
Source: J Med Internet Res. 2024 Oct 1;26:e58831. doi: 10.2196/58831 (PMC11480680; doi:10.2196/58831)
Supplement: Multimedia Appendix 3 [file jmir_v26i1e58831_app3.docx]

# Categories of physician-flagged potentially harmful responses generated by ChatGPT:

### **Overdiagnosis and overtreatment (n=4)**

- Unnecessary referrals: Recommendation of consultations with specialists (e.g., psychologist for positional vertigo) without adequate clinical justification.
- Overdiagnosis: Erroneous diagnosis of a serious condition based on incomplete or inconclusive information (e.g., leukemia based on a single elevated white blood cell count).
- Unnecessary invasive procedures: Suggestion of minimally invasive procedures without comprehensive diagnostic evaluation (e.g., filler injections for plantar fat pad atrophy without further assessment).

### **Underdiagnosis and undertreatment (n=8)**

- Delayed or missed diagnoses: Failing to recognize the necessity for further diagnostic procedures or specialist referrals in cases requiring immediate attention (e.g., unilateral tonsillitis, colon cancer, ventricular tachycardia).
- Delayed treatment: Recommendation of essential vaccinations was withheld due to a mildly elevated risk of herpes infection without clinical symptoms.
- Insufficient urgency: Underestimating the urgency of medical conditions, such as prolonged fever in children, fractures with impaired sensorimotor function, or anemia after major abdominal surgery, leading to potentially harmful delays in seeking professional care.

### **Insufficient patient education (n=3):**

- Failing to provide essential information about potential complications, risk factors, or recommended follow-up care (e.g., risk of malignancy for large gallstones, need for detailed history and clinical examination for asymptomatic thrombocytopenia).

### **Unclear (n=2):**

- No reason stated for categorization as potentially harmful

Two responses were identified as potentially problematic due to an apparent underdiagnosis and undertreatment, as well as an insufficient level of patient education. This resulted in a total of 17 reasons for concern, given that there were 15 potentially harmful responses in total.
